# Usability assessment of a digital tool to enhance person–clinician communication in the memory clinic: An expert evaluation

**DOI:** 10.1177/20552076251365070

**Published:** 2025-08-06

**Authors:** Tanja J. de Rijke, Kyra K.M. Kaijser, Dianne Vasseur, Ellen MA Smets, Leonie N.C. Visser, Marloes Derksen, Thomas Engelsma

**Affiliations:** 1 26066 Amsterdam UMC location University of Amsterdam, Department of Medical Psychology, Amsterdam, the Netherlands; 2 Amsterdam Public Health Research Institute, Personalized Medicine, Amsterdam, the Netherlands; 3 Alzheimer Center Amsterdam, Department of Neurology, Amsterdam UMC, Vrije Universiteit Amsterdam, Amsterdam, the Netherlands; 4 Amsterdam Neuroscience Research Institute, Neurodegeneration, Amsterdam, the Netherlands; 5 Amsterdam Public Health Research Institute, Digital Health, Amsterdam, the Netherlands; 6 Vilans, 99405 the National Centre of Expertise for Care and Support, Utrecht, the Netherlands; 7 Amsterdam Public Health research Institute, Quality of Care, Amsterdam, the Netherlands; 8 Division of Clinical Geriatrics, Center for Alzheimer Research, Department of Neurobiology, Care Sciences and Society, Karolinska Institutet, Stockholm, Sweden; 9 UMC Utrecht, Department of Bioethics & Medical Humanities, Utrecht, the Netherlands; 10 Amsterdam UMC location University of Amsterdam, Department of Medical Informatics, eHealth Living & Learning Lab Amsterdam, the Netherlands

**Keywords:** Dementia, usability, heuristics, communication, human-centred design, evaluation

## Abstract

**Background:**

From a human-centred design perspective, the web-based tool ‘Helder in Gesprek’ (Clear in Conversation) has recently been developed to improve person-centred communication in the memory clinic. The aim was to conduct expert testing to identify potential usability issues prior to user-testing and propose corresponding re-design recommendations.

**Methods:**

The DEMIGNED principles, specifically developed to identify potential usability issues for people with dementia, are used during the expert evaluation. Five experts (*n* = 1 usability, *n* = 2 dementia, *n* = 2 double-experts) used a heuristic evaluation approach applying the DEMIGNED principles to assess the user-interface (navigation structure, lay-out, and interaction) of ‘Helder in Gesprek’. Through deductive and inductive analysis, a unique set of potential usability problems were identified.

**Results:**

Applying the DEMIGNED principles led to the discovery of 42 unique usability problems, related to cognitive principles (*n* = 14), perception principles (*n* = 8), frame of mind principles (*n* = 8), speech and language principles (*n* = 3), or multiple principles (*n* = 9). The mean severity score was 2.32 (SD = 0.85).

**Conclusion:**

We identified several unique usability problems across a variety of DEMIGNED principles before usability testing with actual end-users that need to be addressed during re-design. Designers, researchers, clinicians, and policy makers may use these results to improve the usability of web-based tools, thus improving person-centred communication in the memory clinic for people with cognitive complaints.

## Introduction

The number of people with dementia is estimated to rise from 55 million in 2019 to 139 million by 2050 worldwide, thereby increasing the demand for dementia-related healthcare for example, in memory clinics (see Box 1 for a glossary).^1^ Effective person-centred communication is crucial during consultations, however, a gap is observed where people visiting the memory clinic may not always feel seen or heard.^2–8^ Following a human-centred design approach, we therefore currently develop the Dutch web-based digital tool ‘Helder in Gesprek’ (literally translated: Clear in Conversation).^9^ ‘Helder in Gesprek’ aims to support people visiting a memory clinic to improve person-centred communication (see supplements for an explanation and screenshots; for design and development process, see De Rijke et al., submitted). In order to ensure the usability of ‘Helder in Gesprek’, it is necessary to undertake expert testing to identify and mitigate as many potential usability issues prior to testing with end-users.

Usability problems can arise during the use of an interactive system, e.g. a web-based tool, when the design is not tailored to the needs and capabilities of (specific) users, limiting the effectiveness, efficiency, and satisfaction with a digital tool.^10^ Poor design may lead to decreased acceptability, user engagement, adoption rates, and successful use among users.^11–14^ Specifically in the healthcare setting, usability testing is important to assess whether medical devices and tools are safe to use, user-friendly, and cater to the needs of users.^15^ To assess usability, several usability testing methods exist that involve either expert testing or testing with users. Expert testing encompasses the identification and evaluation of potential usability problems by a group of usability or domain experts using predetermined evaluation criteria, such as usability heuristics or design principles.^16^ By means of expert usability evaluation methods, such as heuristic evaluation, 74–87% of usability problems can already be captured relatively early in the design process allowing for an optimized design during subsequent user testing.^16^ Heuristic evaluation is a form of expert testing in which a small group of evaluators inspect and assess the interface using a list of predetermined usability principles.^10^ Several design principles have been developed aiming to better tailor the design of a system to the needs of people with certain (chronic) medical conditions, such as dementia.^17,18^ Evidence-based design principles offer fundamental concepts that can be seen as foundations for good design and effective interfaces of tools and may inform expert testing, such as heuristic evaluation.^19^ The aim of this study is to identify potential usability problems via expert testing of ‘Helder in Gesprek’ and to provide design recommendations to address these. The case of ‘Helder in Gesprek’ serves as an example of the types of usability problems that may arise, and demonstrates how design principles for people with dementia can be effectively incorporated into expert testing procedures.

## Methods

### Study design: heuristic evaluation using DEMIGNED principles

One of the most common forms of expert testing is a heuristic evaluation (see Box 1 for a glossary of terms).^10^ Heuristic evaluation is a qualitative usability assessment method, during which usability and domain experts test and assess the navigation, lay-out, and interaction of the user interface and structure of a digital tool (see Box 1).^10^ This is done using a set of predefined principles (more overarching guidelines) or heuristics (more specific rules that help to achieve broader design principles).^10^ In healthcare, a commonly used set of heuristics are those by Nielsen and Molich or Zhang et al.^16,20^ However, for this study, we used the recently developed DEMIGNED principles, as these were specifically developed (by TE) to contribute to usable and accessible design of health technologies for people with dementia.^17,18^ For instance, DEMIGNED contains elements on ‘Cognition’ and ‘Frame of Mind’, which are important when designing for people with cognitive complaints and are absent or not as prevalent in existing heuristics. The DEMIGNED principles describe high-level design considerations with actionable design guidelines related to cognition (problems with cognitive abilities resulting in difficulties with understanding, remembering, or interacting with digital systems; e.g. easy navigation to functions and content), perception (problems with the ability to visually and sensorially identifying interface components resulting in difficulties with effective interaction and accessibility; e.g. appropriate system feedback), frame of mind (design elements that consider and support the emotional and psychological well-being of users, ensuring the system is both functional and affirming; e.g. positive feedback for correct action completion), and speech and language (communication difficulties highlighting the need for information that is clear, accessible, and supportive; e.g. understandable words and sentences that feel comfortable).^17,18^ These principles can be applied in the development phase and have previously shown to be promising in identifying more usability problems through expert testing as compared to other sets of heuristics.^21,22^ The outcome of the heuristic evaluation is a list of violations of the applied heuristics, which results in a list of unique usability problems.

Box 1. Glossary of terms

Dementia = Dementia is a syndrome diagnosis that is characterized by cognitive decline due to neurodegenerative changes.^23^

Domain experts = Experts with expertise in specific areas other than usability (e.g. user needs or clinical expertise)

Expert testing = The aim of expert testing is to uncover ‘*potential usability problems by having evaluators inspect a user interface with a set of guidelines, heuristics or questions in mind or by performing a step-wise approach, derived from general knowledge about how humans process through tasks.’*^10^

Heuristics = ‘*A set of recognized usability principles that refer to common properties of usable systems’*^10^

Heuristic evaluation = a method to evaluate the usability of a system during which ‘*a small set of evaluators inspects a system and evaluates its interface against a list of recognized usability principles—the heuristics*.’^10^

Heuristic violation = A heuristic violation occurs when a system fails to adhere to a heuristic.

Master list = A comprehensive compilation of usability problems that were identified by multiple evaluators during their independent assessments of the tool or system that is being evaluated.^4,21,24^

Memory clinic = Memory clinics are multidisciplinary clinics aiming for an early and timely diagnosis and support for people with cognitive complaints.^25^ People visiting the memory clinic may comprise people living with dementia, people with mild cognitive impairment, in which early cognitive decline can be clinically observed, and people with subjective cognitive decline, in which people experience cognitive decline without objective clinical cognitive decline.^26^

Nielsen severity score = The Nielsen Severity Score is a widely used scale that assesses to the severity of usability problems.^2,6,8,16,27,28^ Scores range from ‘*0) I do not agree that this is a usability issue at all, 1) Cosmetic issue only: need not to be fixed unless extra time is available on project, 2) Minor usability issue: fixing this should be given low priority, 3) Major usability issue: important to fix, so should be given high priority, or 4) Usability catastrophe: imperative to fix this before the product can be released*’.^16,28^

Usability = ‘*The extent to which a system, product, or service can be used by specific users to achieve goals with effectiveness, efficiency, and satisfaction in a specified context of use*’^29^

Usability experts = Experts with expertise in usability

Usability problems = Problems that arise during use when the design is not tailored to (specific) users and negatively affect the effectiveness and efficiency of a digital tool as well as the satisfaction with the tool.^10,27^

### Participants and procedures

Five experts (TE, MD, DV, KK, TR) independently conducted the heuristic evaluation. Previous research has shown that 3–5 experts can capture 74–87% of all usability problems.^30^ Two participants (TE, DV) were double experts in both human-technology interaction and dementia, whereas the other three were domain experts in medical informatics (MD) or dementia (KK, TR) (see Table 1 for characteristics of experts). Prior to testing of the website and subsequent usability evaluation, TE provided training on the application of the DEMIGNED principles during heuristic evaluations, comprising an introductory lecture explaining heuristic evaluations and the DEMIGNED principles, followed by a practice assignment to identify potential usability problems. Results hereof were discussed and additional explanations were given when needed. The heuristic evaluations took place in the Amsterdam UMC, location AMC, in the Netherlands in August 2024.

**Table 1.** Overview of characteristics of experts participating in the heuristic evaluation.

	Research expertise	Gender	Experience in years^a^	Degree
Expert 1 (TE)	Medical informatics; eHealth; usability engineering; design for people living with dementia	Male	7 years	PhD
Expert 2 (MD)	Digital inclusivity; culturally-diverse and underrepresented populations; eHealth; medical informatics	Female	7 years	PhD
Expert 3 (DV)	Industrial design; human-technology interaction; implementation; digital health; dementia	Female	4 years	MSc
Expert 4 (KK)	Medical psychology; psychobiology; policy; dementia	female	1,5 years	MSc
Expert 5 (TR)	Medical psychology; health sciences; dementia	female	3,5 years	MSc

a Experience with topic of expertise.

All experts independently navigated through the entire web-based ‘Helder in Gesprek’ tool, completing the tasks available in the tool whilst assessing the navigation, layout, and interaction for violation of the DEMIGNED principles (see Table 2). When a violation was detected, the corresponding location and violated DEMIGNED principle(s) were noted in an Excel sheet by all experts independently. Usability problems can be related to multiple DEMIGNED principles, allowing the experts to report more than one principle for a unique usability issue.^14^ Hereafter, a Nielsen severity ranking score was given to each particular violation by all experts independently.^16^ After individual assessments of all experts, all identified usability problems were combined in one master list by TR, after which duplicate problems were removed, mean severity scores were calculated, and results were discussed to achieve team consensus (see Supplementary Tables 1–5 for master list).

**Table 2.** Overview of tasks performed during heuristic evaluation.

1. On the website you can answer items about yourself with yes or no. Can you please answer these items for me?
2. If someone wants to change some of the items you can. Can you please change an answer to the items for me?
3. In the next step you can give an explanation the items. Can you please give an explanation of four items for me?
4. In the next step, you can summarize the topics you would like to discuss with your healthcare provider. Can you please make a summary for me?
5. In the next step, you can make a summary and download it or send it to your memory clinic. Can you please send your summary to your own e-mail address for me?

### Data analysis

**Figure 1.**
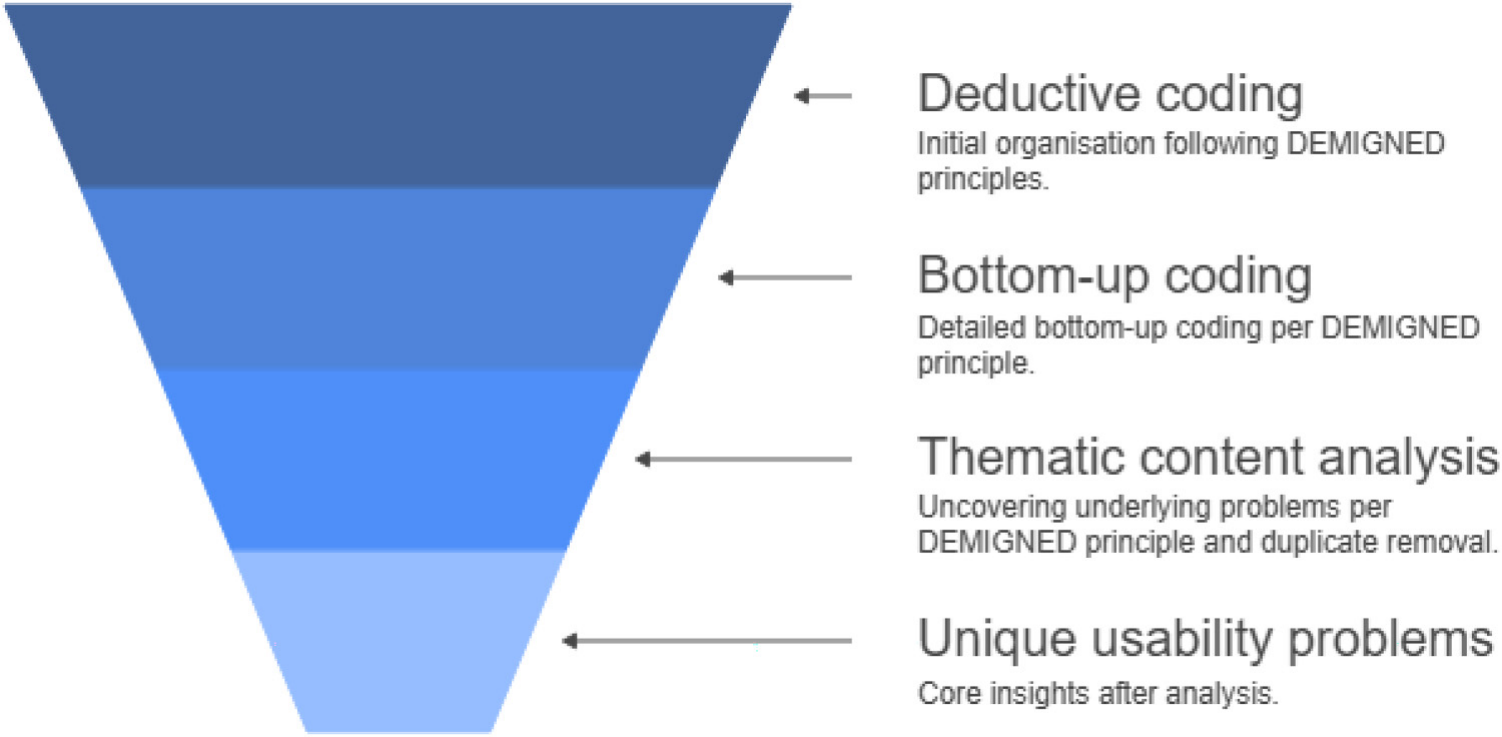
Overview of the coding process.

The master list with usability problems was first analysed (TR) using deductive coding based on themes (cognition, perception, frame of mind, and speech and language) of the DEMIGNED principles (see Figure 1). Per theme, bottom-up coding was then performed, using a thematic content analysis, to uncover the underlying usability problems per DEMIGNED principle. Based on this thematic content analysis, duplicate themes were merged, which led to the final number of unique usability problems. To ensure reliability, two other researchers (KK and TE) independently checked all steps of analysis. When any conflicting issues arose, these were discussed until consensus was reached and the assignment of the usability problem was adjusted accordingly.

A mean Nielsen severity score was calculated for each unique usability issue to mitigate the potential for extreme viewpoints given the relative novelty of the DEMIGNED principles.^14^ To highlight the potential usability problems with higher severity scores, the results will describe only those usability problems with a Nielsen severity score above the mean (e.g. if the mean severity score of the entire sample is three, only usability problems having a severity score of three or higher will be reported on). However, the full master list is available in Supplement 2. Finally, based on the findings, a design brief was created supplemented with design recommendations as reported in literature.

### Ethics

The Medical Ethics Committee of the Amsterdam UMC, location AMC, approved the ‘Helder in Gesprek’ study (METC number W22_377 # 22.449). All experts provided informed consent prior to participation.

## Results

The heuristic evaluation resulted in a total of 42 unique usability problems (see Supplementary Tables 1–5 for master list). Of these, fourteen (14/42; 33.3%) could be related to ‘cognition’, mainly centring on navigation. For example, users experienced unclarity about how to transition from answering items to follow-up questions that depended on their earlier responses. Eight (8/42; 19%) problems could be themed as ‘perception’, comprising system feedback problems along with some problems related to clickability, compartmentalization, elements, and colour use, such as a potential lack of colour contrast between a button and background. For ‘Frame of mind’, eight (8/42; 19%) problems were identified, including problems with content, preferences, support, and positive feedback, such as the need for a neutral answer option besides ‘yes’ and ‘no’ for all items in situations where users do not know what to answer. Three (3/42; 7.1%) problems were categorized as ‘Speech and language’, i.e. problems related to understandability and some options for user input, such as inconsistency in the button texts and other texts on the website. Nine (9/42; 21.4%) usability problems were considered to be a mix of themes, such as ‘Frame of mind’ combined with ‘Cognition’ or ‘Perception’. The mean severity of usability problems was rated 2.32 (SD = 0.85; observed min = 0, max = 4). Below, a concise descriptive overview is given, categorized by DEMIGNED themes and principles (see Table 3 and Supplementary Tables 1–5 for master list).

**Table 3.** Concise overview of identified usability problems related to the DEMIGNED themes and principles.

Violated DEMIGNED Theme	Violated DEMIGNED principle(s)	Bottom-up theme usability problem	Problem description	# of times identified^a^	Mean Nielsen severity
Cognition	C-Navigation	Obligation to fill in all statements	It is imperative that all items are answered with yes or no (step 1) before progression to a next step, in which users may provide additional information for items that were previously answered with yes (step 2). In the event of a missed item, users will need to scroll back and locate the item manually, which may cause frustration and prevent task completion.	3	3,7
	C-Monitoring	No overview of previously filled in items	In step 3, users can provide a summary based on their previous answers. However, an overview of the user's responses during previous steps is lacking. This requires users to recall their responses, potentially leading to incomplete information and resulting in cognitive overload and frustration.	4	3,3
	C-Navigation	No navigation support between steps	It is unclear to users how to navigate between consecutive steps and pop-up questions, which may be confusing and frustrating.	4	3,1
	C-Navigation	Automatic exit email address	If users click on an email address, they will automatically be redirected from the website, causing them to be unable to return to the website, which may be confusing and frustrating and prevent task completion.	1	3
	C-Tutorials	No information on how to receive the output	Instructions on how to receive the output from the tool (a summary made by the user containing the most important topics that they want to discuss with their clinician) are lacking, which may cause confusion and prevent users from achieving the goal of the tool.	1	3
	C-Navigation; C-Tutorials	No feedback to scroll	There is no system feedback to notify users they have to scroll down through the items, which might not be clear to users.	11	2,5
	C-Navigation	No intuitive home and question mark button	The function of the ‘home’ button, ‘question mark’ button and the location of the ‘back to overview’ button are not intuitive and lack informative text (e.g. ‘home’ or ‘back’). Also, users cannot ask a question or see frequently asked questions underneath the ‘question mark’ button. For all, it is unclear what page the user will be referred to, which may be confusing for users.	15	2,7
Frame of mind	F-Content	Missing answer option	Users may need an answer option such as ‘I don't know’ for all items in situations where they do not know what to answer. This may induce worries or frustration for the user.	1	3
	F-Support	No automatic ordering	In step 3, users have to create a summary by selecting items that they previously indicated to be most important to them. There is no automatic ordering in step 3 during the selection of items, which may be frustrating for users.	1	3
Mixed	C-monitoring; C-Navigation; C-Tutorials; F- Support	No indication unanswered statement	If a user forgets to answer an item with yes or no and is referred back to complete this particular item, it is unclear for the user why they are referred back and cannot proceed, which may be frustrating for users and prevent task completion	1	4
	C-Monitoring; F-Positive feedback	Possibility of empty summary	If users do not fill in any additional information when items are answered with ‘yes’, they end up with an empty summary, which may result in people concluding that they did not fill in the tool successfully and therefore start all over or drop out.	1	4
	C-Abilities; F-support	No confirmation of email address	After clicking on ‘send summary’ to submit output from the tool to their email address, users are not asked to confirm their email address. This may lead to unwanted situations, which may result in data leaks or people concluding that they were not successful in sending the summary and therefore start all over or drop out.	1	4
	F-Content; C-Abilities	Cognitive overload	For items that users answered with yes, they may fill in additional questions. These additional questions present a multitude of answer options without repeating the item to which the answers apply. Consequently users have to remember the items, which may be overwhelming and frustrating, potentially leading to cognitive overload.	5	3,6
	F-Positive feedback, P-System feedback	No preview or print option summary	There is no preview or print option for the summary that the user can send to the clinician, which may be frustrating for some users as they have to remember what was in their summary and why.	4	3
	F-Positive feedback. P-Click ability	Touchscreen response	Ticking multiple choice options does not always work well, for instance, due to button sizes (especially the ‘other’ option), which may be frustrating for users.	1	3
	F-Content; S-Understandability	Irrelevant and unclear content	Changes in the written text are needed to improve clarity, relevance, and alignment with B1 language level, since as it stands may be confusing or frustrating for users.	49	2,6

^a^ An overview of all usability problems with a Nielsen severity score of >2.32. For the master list with all identified, unique usability problems see Supplementary Tables 1–5.

The list of unique usability problems and DEMIGNED principles, supplemented with design recommendations as reported on in relevant literature, resulted in the design brief as depicted in Table 4.

**Table 4.** Design brief for designers and researchers, clinicians, and/or policy makers for the development of web-based tools for communication or decision-making designed for people with dementia based on the list with unique usability problems.

DEMIGNED principle	Design recommendation
Cognition	*Designers:* Focus on improving navigation guidance between pages/steps, use appropriate, clear and correctly sized icons, and incorporate simple tutorials.^31–35^
	*Researchers, clinicians, and/or policy makers:* Create the content for integrated or separate tutorials and be prepared to provide training and/or hands-on personalized support when needed.^31^
Perception	*Designers:* Improve system feedback and check for screen jumps, accessible colour contrast, and auditory feedback when clicking on something.^14,31,32^
	*Researchers, clinicians, and/or policy makers:* Align with evidence-based practices for supporting users with impaired perception, evaluate the effectiveness of the implemented mechanisms, and ensure the system complies with relevant accessibility regulations.^36^
Frame of Mind	*Designers:* Build in positive feedback, provide options for personalization, provide the option that actions can be undone, and, where possible, use automatic ordering.^31,33,34,37,38^
	*Researchers, clinicians, and/or policy makers:* Explore relevant options for personalization together with the users.^39^
Speech and Language	*Designers:* Create an option for user input (such as speech-to-text function or obligatory text input) and check for understandability and consistency of texts and buttons.^34^
	*Researchers, clinicians, and/or policy makers:* Provide accessible texts that are co-developed or pilot tested on clarity with users.^14,31,40^
Mixed	*Designers:* Develop a clear and intuitive menu and help function (e.g. search bar), provide progress indication and highlight forgotten questions, build in a check so users cannot continue to the next step if they did not answer the necessary questions, do not provide a multitude of options based on a previous questions that users have to recall, provide intuitive text lay-out and placation, and provide the option for a preview or print of the end-result.^32,34,35,41^
	*Researchers, clinicians, and/or policy makers:* Provide tangible, positive, non-paternalistic, empowering, accessible texts in small pieces that are co-developed or pilot tested on clarity and cognitive overload with users. Offer training prior to use to all, and personalized support when needed.^14,31,32,34^

## Discussion

We found 42 unique potential usability problems with a mean severity score of 2.32, indicating moderate usability problems. These usability problems were found across several DEMIGNED themes; however, more severe usability problems were identified among the themes ‘Cognition’ and ‘Frame of Mind’. The most common violated design principle comprised navigation, indicating room for design improvements. A previous study that assessed a mobile website using the DEMIGNED principles during heuristic evaluation also found a majority of violations centring on navigation problems, which was confirmed in their subsequent end-user testing.^14^ A recent systematic review on web-based tools for communication or decision-making designed for people with dementia also found navigation as one of the main design recommendations.^31^ The authors, for instance, recommended streamlining information access when it comes to navigation, for instance, via employing efficient search functions.^31^ Other design recommendations that seem to overlap with our findings comprise enhancing support by offering (pre-)training and tailored support. However, we did not identify major usability problems related to visual appearance or delivery of content, although problems could occur during user-testing.

We demonstrated that applying the DEMIGNED principles in expert usability evaluations is feasible to capture potential usability problems of a web-based digital tool in the context of dementia. Moreover, the focus of identified usability problems was centred on the themes ‘Cognition’ and ‘Frame of Mind’, which are not as prevalent in existing sets of heuristics, highlighting that DEMIGNED is able to capture additional relevant usability problems for people with dementia.

A strength of this study was that with two double experts and three domain experts, each with their own perspective and expertise, the consensus meetings led to the application of multiple design principles for a single usability issue. Having expert evaluators with diverse backgrounds is recommended as this enables to identify a broad and nuanced range of usability problems, which is reflected in our study by the relatively large proportion of usability problems based on a combination of several DEMIGNED principles. This study also has limitations worth mentioning. First, usability issues were reported as overall frequencies rather than the number of evaluators, which is not common within heuristic evaluations. However, this approach provides a more comprehensive view of the most prevalent issues, highlighting patterns across evaluators, and ensuring that frequently identified problems are prioritized for improvement. Second, this study only reports findings from expert testing, representing potential and therefore, more subjective usability problems. Expert-testing is not meant to substitute user-testing, but is rather meant to be done complimentary and prior to user-testing. This allows us to address severe usability problems before including the actual users in usability testing. The results from this study will be used to guide future user testing with ‘Helder in Gesprek’.

As for practical implications, the results from this and previous studies emphasize the importance of incorporating design aspects to support navigation. Examples of strategies to improve navigation comprise audio cues, implementing a combination of linear navigation, and a help text to guide users through the web-based tool.^31^ Other design elements to consider include clarity regarding user expectations, scrolling support, accessible texts, and avoidance of cognitive overload. Incorporating these design elements is not only relevant when designing web-based digital tools for people with dementia, but it may result in improved accessibility and satisfaction for all users from a universal design perspective.^42,43^

## Conclusion

This heuristic evaluation identified usability problems across several DEMIGNED themes. Designers, researchers, clinicians, and policy makers may use these results to improve the usability of web-based tools, thus improving person-centred communication in the memory clinic for people with cognitive complaints.

## Supplemental Material

sj-docx-1-dhj-10.1177_20552076251365070 - Supplemental material for Usability assessment of a digital tool to enhance person–clinician communication in the memory clinic: An expert evaluation Supplemental material, sj-docx-1-dhj-10.1177_20552076251365070 for Usability assessment of a digital tool to enhance person–clinician communication in the memory clinic: An expert evaluation by Tanja J. de Rijke, Kyra K.M. Kaijser, Dianne Vasseur, Ellen MA Smets, Leonie N.C. Visser, Marloes Derksen and Thomas Engelsma in DIGITAL HEALTH
