# Supplementary material for: Usability assessment of a digital tool to enhance person–clinician communication in the memory clinic: An expert evaluation
Source: Digit Health. 2025 Aug 6;11:20552076251365070. doi: 10.1177/20552076251365070 (PMC12329271; doi:10.1177/20552076251365070)
Supplement: sj-docx-1-dhj-10.1177_20552076251365070 - Supplemental material for Usability assessment of a digital tool to enhance person–clinician communication in the memory clinic: An expert evaluation [file sj-docx-1-dhj-10.1177_20552076251365070.docx]

# Supplementary materials for manuscript: ‘Usability assessment of a digital tool to enhance person-clinician communication in the memory clinic: an expert evaluation’

**Supplement 1: study case description of the web-based prototype of ‘Helder in Gesprek’**


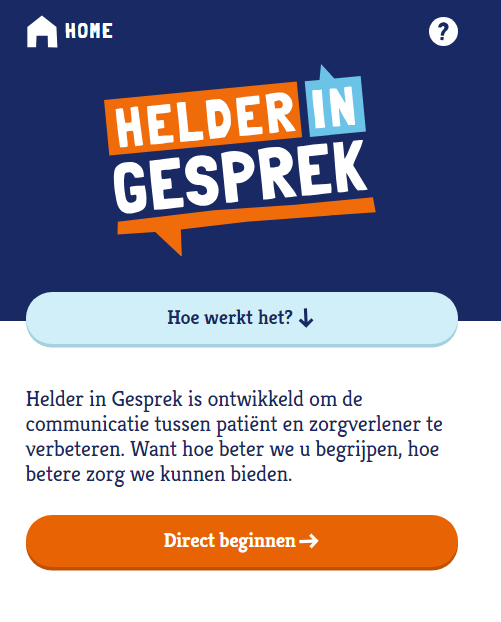


‘Helder in Gesprek’ was developed based on a co-research and co-design project conducted in 2023-2025 in the context of the ABOARD project (©2024-ABOARDxAmsterdamUMC. All rights reserved.).^20^ The aim of the tool is to improve person-centred communication in the memory clinic via supporting people visiting the memory clinic to think about topics that they would want their clinician to know about them. The tool starts with a general introduction, followed by 20 items (e.g. ‘I live alone’, ‘I find it hard to ask for help’, or ‘I want to prepare myself for the future’) (see Supplementary Figure 1 and 2). People can answer the items with either ‘yes’ or ‘no’. If someone answers an item with ‘yes’, follow-up questions will be asked, for instance, comprising prompts on why someone finds this topic important and how important they find this topic to discuss with their memory clinic clinician. At the end, an overview is generated containing three topics that the user considers most important to discuss with their memory clinic clinician.

Supplementary Figure 1 – Overview of the landing page of ‘Helder in Gesprek’ (©2024-ABOARDxAmsterdamUMC. All rights reserved.)


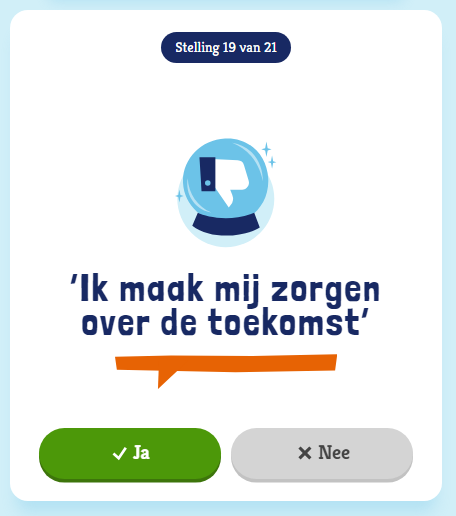

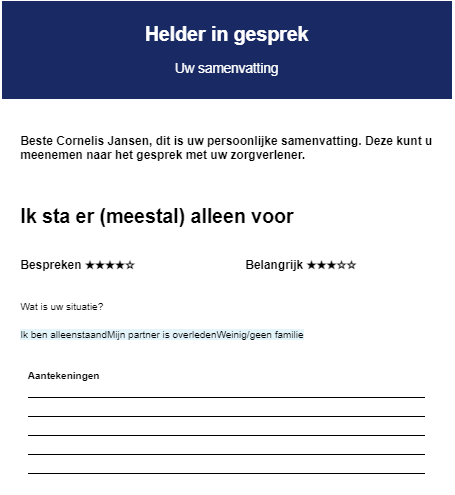

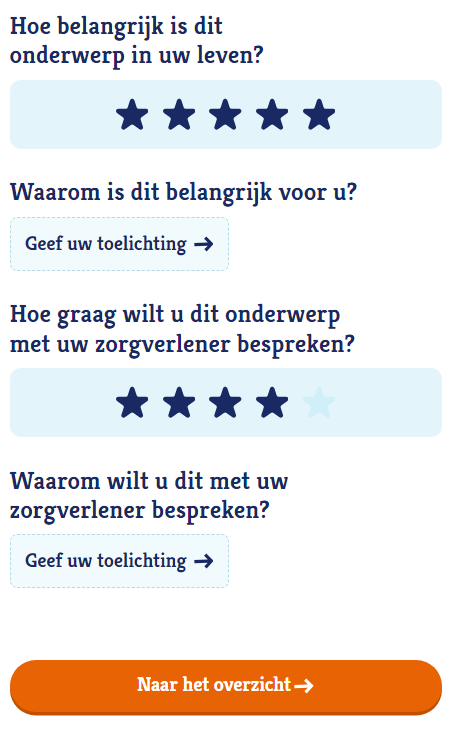


Supplementary Figure 2 - Exemplary questions on the website and summary. Data in these pictures are based on an imaginary use case (©2024-ABOARDxAmsterdamUMC. All rights reserved.)

**Supplement 2: Master lists of all identified usability problems**

**Table S1. Master list of identified usability problems related to the violated DEMIGNED theme ‘Cognition’**

| **Violated DEMIGNED principle(s)** | **Bottom-up theme usability problem** | **Problem description** | **# of times identified** | **Mean Nielsen severity** |
| --- | --- | --- | --- | --- |
| C-Navigation | Obligation to fill in all items | It is imperative that all items are answered with yes or no (step 1) before progression to a next step, in which users may provide additional information for items that were previously answered with yes (step 2). In the event of a missed an item, users will need to scroll back and locate the item manually, which may cause frustration and prevent task completion. | 3 | 3,7* |
| C-Monitoring | No overview of previously filled in items | In step 3, users can provide a summary based on their previous answers. However, an overview of the user’s responses during previous steps is lacking. This requires users to recall their responses, potentially leading to incomplete information and resulting in cognitive overload and frustration. | 4 | 3,3* |
| C-Navigation | No navigation support between steps | It is unclear to users how to navigate between consecutive steps and pop-up questions, which may be confusing and frustrating. | 4 | 3,1* |
| C-Navigation | Automatic exit email address | If users click on an email address, they will automatically be redirected from the website, causing them to be unable to return to the website, which may be confusing and frustrating and prevent task completion. | 1 | 3* |
| C-Tutorials | No information on how to receive the output | Instructions on how to receive the output from the tool (a summary made by the user containing the most important topics that they want to discuss with their clinician) are lacking, which may cause confusion and prevent users from achieving the goal of the tool. | 1 | 3* |
| C-Navigation | No intuitive home and question mark button | The function of the ‘home’ button, ‘question mark’ button and the location of the ‘back to overview’ button are not intuitive and lack informative text (e.g., “home” or “back”). Also, users cannot ask a question or see frequently asked questions underneath the ‘question mark’ button. For all, it is unclear what page the user will be referred to, which may be confusing for users. | 15 | 2,7* |
| C- Navigation; C-Tutorials | No feedback to scroll | There is no system feedback to notify users they have to scroll down through the items, which might not be clear to users. | 11 | 2,5* |
| C-Navigation | Flow summary redo all steps | If the 'See summary' button is selected, the previous steps will need to be repeated before the summary will be displayed. This may cause frustration for users, who may expect to see the summary immediately. | 1 | 2 |
| C-Navigation; C-Tutorials | Too attractive ''Start immediately'' button | There is a tendency to hit the ‘Start immediately’ button right away, missing information and instructions for users potentially resulting in unclarity later on. | 2 | 2 |
| C-Navigation; C-Tutorials | Two buttons how does this work | There are two buttons containing information on 'How does it work?'. Users may get confused because there are two buttons on the same topic and may start to wonder which of these provides an answer to their question. | 1 | 2 |
| C-Icon use | Suggestive icons or bars | The orange bar of the logo subtly suggests an answer when completing items, and a check icon indicates a positive response, while a cross indicates a negative one. These elements potentially influence users. | 1 | 1,5 |
| C-Icon use | Size of icons | In the overview, some green check icons are larger than others even though they mean the same, which may cause confusion for users. | 3 | 1 |
| C-Icon use | No intuitive icon | The icon's choice of stars and the left-arrow direction may appear counterintuitive, which could potentially cause confusion among users. | 2 | 1 |
| C-Tutorials | No referral button to cookies page | There is no option to read more about cookies; they only appear later on the home page if the user has already accepted cookies, which may be a desirable feature for users. | 1 | 1 |

**Notes. Also shown in Table 3. in the manuscript.*

**Table S2. Master list of identified usability problems related to the violated DEMIGNED theme ‘Frame of Mind’**

| **Violated DEMIGNED principle(s)** | **Bottom-up theme usability problem** | **Problem description** | **# of times identified** | **Mean Nielsen severity** |
| --- | --- | --- | --- | --- |
| F-Content | Missing answer option | Users may need an answer option such as 'I don't know' for all items in situations where they do not know what to answer. This may induce worries or frustration for the user. | 1 | 3* |
| F-Support | No automatic ordering | In step 3, users have to create a summary by selecting items that they previously indicated to be most important to them. There is no automatic ordering in step 3 during the selection of items, which may be frustrating for users. | 1 | 3* |
| F-Positive feedback | Progress bar implies not finished | In the second step of the process, users are required to enter a minimum of four additional questions in response to items before they can continue. Upon completion of this step, users will return to the overview page, where a progress bar shows how many questions are left unanswered, which may confuse people into thinking that they have to continue to fill in all answers. | 3 | 2,3 |
| F-Positive feedback | Minimal visual feedback filled in | In step 2, there is little visual feedback when additional information is entered on an item, which may cause confusion and frustration for the user since they may forget whether or not they already answered the question or not. This may lead to cognitive overload. | 5 | 2,2 |
| F-Positive feedback | Expectations finish button | The text in combination with the 'Finish' button can create a misleading impression for users, as the text suggests that tasks still need to be completed, even though there is a finish button. It may be unclear for users if they actually finished the entire tool or whether they still need to do something. | 1 | 2 |
| F-Support | Clicks cannot be undone | Once a star or answer option has been selected in the items, it cannot be deselected, which may cause frustration for users. | 2 | 2 |
| F-Preferences | No personalisation options | There is no option to personalise the text, for example by adjusting the font sizes or selecting 'other' to indicate that the text should not be sent automatically to the clinician. This may cause frustration, mistrust and inaccessibility for some users. | 5 | 1,7 |
| F-Preferences | Unclarity about data privacy | More clarity is needed regarding the anonymity and having to fill in personal details, as it may not be clear to users who can see their answers and could potentially cause concern. | 1 | 1 |

**Notes. Also shown in Table 3. in the manuscript.*

**Table S3. Master list of identified usability problems related to the violated DEMIGNED theme ‘Perception’**

| **Violated DEMIGNED principle(s)** | **Bottom-up theme usability problem** | **Problem description** | **# of times identified** | **Mean Nielsen severity** |
| --- | --- | --- | --- | --- |
| P-Elements | Screen jumps | There are some screen jumps making the text unreadable, which harms the usability for users. | 3 | 2,3 |
| P-System feedback | No confirmation of sending email | There is no confirmation screen displayed after the email has been sent. It is possible to continue to resend an email. It is not clear to the user that this task has already been completed, which may lead to frustration or worries. | 3 | 2,3 |
| P-System feedback | Cannot decline cookies | It is not possible to decline cookies, which may cause frustration for end-users. Additionally, the boxes for functional and analytical cookies appear to be clickable, but are in fact unclickable, which may also cause frustration and confusion for users. | 2 | 2,3 |
| P-System feedback | Check icon appears even with incomplete questions in step 2 | In instances where not all comments or stars have been filled in, an item is still ticked off (green tick) without prompting the user to confirm its accuracy. This may be misleading, and the user may assume that they have completed all fields. | 2 | 2 |
| P-System feedback | No warning of having to fill in at least 4 times 'yes' until you are at step 2 | If users answer all items with 'no' in step 1, users can continue until the end of step 2 without a notification. However, further in the tool, you cannot continue or get a summary if you only clicked no, so some people may have to go back to the start. This may be frustrating for users who have to go all the way back. | 1 | 2 |
| P-Clickability | No auditory feedback | There is no auditory confirmation when users click on a selection. This may cause unclarity for users whether they clicked correctly or not. | 2 | 1,5 |
| P-Color use | Color contrast and color of button | It is not clear whether the colour contrast between the filled-in and unfilled sections, as well as the stars and background, is sufficient for users. There is a possibility that this may not be sufficient colour contrast, which could potentially result in confusion and frustration for users. Additionally, the buttons 'agree', 'to the overview', and the bar at the top of the website are red, which may be alarming for users. | 3 | 1,3 |
| P-System feedback | Missing/confusing text support loading | Upon initial loading of the website, a light blue moving circle is the only visible element. The text-based loading indicator is not present, which may cause confusion for end users. Additionally, upon completion of page loading, a message stating 'These were all the items' is displayed on every page, potentially leading to ambiguity in some instances. This may result in confusion for users. | 2 | 1,3 |

**Table S4. Master list of identified usability problems related to the violated DEMIGNED theme ‘Speech and Language’**

| **Violated DEMIGNED principle(s)** | **Bottom-up theme usability problem** | **Problem description** | **# of times identified** | **Mean Nielsen severity** |
| --- | --- | --- | --- | --- |
| S-Understandability | Unclear who summary is send to | The button 'send my information' does not specify who it will be sent to, which may cause confusion for users. | 1 | 2 |
| S-Understandability | Inconsistency button texts and copy | The text (on buttons) sometimes lacks consistency, which is now not always consistent and may be confusing for users. | 5 | 1,9 |
| S-User input | Inconsistency button texts and copy | There is no text-to-speech function available, which may be inaccessible for users. | 2 | 1,5 |

**Table S5. Master list of identified usability problems related to multiple violated DEMIGNED themes**

| **Violated DEMIGNED principle(s)** | **Bottom-up theme usability problem** | **Problem description** | **# of times identified** | **Mean Nielsen severity** |
| --- | --- | --- | --- | --- |
| C-Monitoring; C-Navigation; C-Tutorials; F- Support | No indication unanswered items | If a user forgets to answer an item with yes or no and is referred back to complete this particular item, it is unclear for the user why they are referred back and cannot proceed , which may be frustrating for users and prevent task completion | 1 | 4* |
| C-Monitoring; F-Positive feedback | Possibility of empty summary | If users do not fill in any additional information when items are answered with ‘yes’, they end up with an empty summary, which may result in people concluding that they did not fill in the tool successfully and therefore start all over or drop out. | 1 | 4* |
| F-Support; C-Abilities | No confirmation of email address | After clicking on ‘send summary’ to submit output from the tool to their email address, users are not asked to confirm their email address. This may lead to unwanted situations, which may result in data leaks or people concluding that they were not successful in sending the summary and therefore start all over or drop out. | 1 | 4* |
| F-Content; C-Abilities | Cognitive overload | For items that users answered with yes, they may fill in additional questions. These additional questions present a multitude of answer options without repeating the item to which the answers apply. Consequently users have to remember the items, which may be overwhelming and frustrating, potentially leading to cognitive overload. | 5 | 3,6* |
| F-Positive feedback; P-System feedback | No preview or print option summary | There is no preview or print option for the summary that the user can send to the clinician, which may be frustrating for some users as they have to remember what was in their summary and why. | 4 | 3* |
| F-Positive feedback; P-Click ability | Touchscreen response | Ticking multiple choice options does not always work well, for instance, due to button sizes (especially the ‘other’ option), which may be frustrating for users. | 1 | 3* |
| F-Content; S-Understandability | Irrelevant and unclear content | Changes in the written text are needed to improve clarity, relevance, and alignment with B1 language level, since as it stands may be confusing or frustrating for users. | 49 | 2,6* |
| P-Elements; C-Icon use | Text lay-out and placation | There are several issues with the text layout that could potentially reduce the clarity for the users. The text on the development of the tool is quite central on the page, which is usually more below, and its relevance to end-users may be questionable here, potentially resulting in cognitive overload. The placement of the placation text is also a concern. The placement of the text that you have to fill in extra information for at least 4 items is too low on the page, which may be unclear for users. The user does not see that system feedback at the bottom of the page (the number of items) changes when items are selected, which may also be unclear for users. | 7 | 2,1 |
| F-Support; C-Abilities | No dropdown option contact details | There is no menu or overview to facilitate the quick retrieval of contact details, which may be frustrating for users. | 1 | 2 |

**Notes. Also shown in Table 3. in the manuscript.*
